# A Study on Pregenomic RNA and Factors Related to Hepatitis B Virus Infection Based on Real World

**DOI:** 10.3389/fpubh.2022.856103

**Published:** 2022-06-15

**Authors:** Hao-Zhen Yan, Zhi-Hao Huang, Xu-Guang Guo, Ting-Ting Peng, Li-Li Yang, Chong-Wen Liu, Shi Ou-Yang

**Affiliations:** ^1^Department of Infectious Diseases, The Fifth Affiliated Hospital of Guangzhou Medical University, Guangzhou, China; ^2^School of Public Health, Department of Preventive Medicine, Guangzhou Medical University, Guangzhou, China; ^3^Department of Clinical Laboratory Medicine, The Third Affiliated Hospital of Guangzhou Medical University, Guangzhou, China

**Keywords:** pregenomic RNA, hepatitis B virus, FIB-4, tenofovir, entecavir

## Abstract

**Objective:**

This article aims to study the influencing factors of pgRNA and its change magnitude based on the real world.

**Methods:**

A total of 421 patients who were tested for pgRNA were selected. According to the baseline data, the subjects were divided into negative and positive groups. The Chi-square test and logistic regression were used to analyze the influencing factors of pgRNA status. Based on the follow-up data, the rank-sum test and linear regression were used to analyze the influencing factors of pgRNA change magnitude.

**Results:**

A total of 153 (36.3%) of the 421 subjects were pgRNA-negative and 268 (63.7%) were pgRNA-positive. Logistic regression analysis showed that positive HBV DNA (OR: 40.51), positive HBeAg (OR: 66.24), tenofovir treatment (OR: 23.47), and entecavir treatment (OR: 14.90) were the independent risk factors for positive pgRNA. Univariate linear regression showed that the pgRNA change magnitude of patients treated with entecavir was higher than that of patients treated with tenofovir. Multivariate linear regression showed that age was an independent factor influencing pgRNA change magnitude.

**Conclusions:**

The pgRNA of patients who were young, female, HBV DNA-positive, high-HBsAg, HBeAg-positive is higher than the detection line. HBV DNA and HBeAg are the independent risk factors of positive pgRNA. Different antiviral regimens and disease stages have significantly different effects on pgRNA status. There was a significant correlation between pgRNA and FIB-4, suggesting that pgRNA is related to liver fibrosis. The decrease in pgRNA was greater in young patients than in non-young patients. The decrease in pgRNA was greater in patients treated with tenofovir than in patients treated with entecavir.

## Introduction

Hepatitis B virus (HBV), as a kind of global communicable disease, is prevalent in Asian and African countries especially. There are ~257 million chronic HBV infections in the world. It is estimated that there are currently about 70 million chronic HBV infections in China, including about 20 to 30 million patients with chronic hepatitis B (CHB) (1).

It (2, 3) is found that during the life circle of hepatitis B virus, HBV cccDNA (covalently closed circular DNA) in liver cells, as the most important template of the HBV RNA coding, is not only the beginning of virus replication but also the key factor of persistent infection and recurrence. The current therapies are proved to exert less influence on cccDNA, the long-term existence of which is an important reason for chronic HBV (4). Theoretically, the cccDNA level should be the most direct index of prognostic (5). However, due to (6–8) its uneven distribution and damage caused by invasive examination, the application of cccDNA is limited. Conversely, the examination of HBV pgRNA (pregenomic RNA) is simple and non-invasive for the sampling, thus more acceptable to patients (9).

It (4) is proved that pgRNA not only is the direct transcription product of cccDNA but also can encode HBV polymerase which can convert pgRNA into rcDNA. The rcDNA can be then repaired to form HBV cccDNA (4). The research (10) also suggests that pgRNA is positively correlated with cccDNA. It is obvious that pgRNA has a chance to replace cccDNA to play a key role in antiviral therapy.

The current evaluation of antiviral therapy efficacy depends on HBV DNA (11, 12). However, after the reverse transcription of pgRNA is controlled, the serum HBV DNA levels of CHB decrease, whereas the levels of cccDNA decrease less. Below detection limit HBV DNA only means the reverse transcription is restrained, instead of the indicator of withdrawal, because cccDNA may still express (11, 12). Theoretically, even one replicate-capable cccDNA in liver cells can lead to recurrence once antiviral therapy is stopped (11). Therefore, the removal of cccDNA is considered important to eradicate persistent HBV infection (3). American Association for the Study of Liver Diseases (AASLD) and European Association for the Study of the Liver (EASL) also defined “HBV completely cured” as “HBsAg in serum is undetectable and HBV DNA is eradicated, including the eradication of cccDNA and integrated HBV DNA in the liver.” Therefore, apart from HBV DNA, it is necessary to follow cccDNA to estimate the prognosis of patients with HBV.

Furthermore, nucleos(t)ide drugs can only block the reverse transcription of pgRNA without affecting its production (13, 14). Even when HBV DNA synthesis is blocked, cccDNA can still synthesize new HBV particles in the HBV RNA virus-like particle mode (15). Therefore, when pgRNA cannot be detected in serum, cccDNA in the liver has disappeared or stopped transcribing, which means the medication can be stopped (10, 16).

It is necessary to conduct epidemiological studies on pgRNA, since not many of those have been published currently.

## Objects and Methods

### Patients and Data Collection

Eligible participants were adults with confirmed hepatitis B virus infection and complete case-related information, who have detected pgRNA at The Fifth Affiliated Hospital of Guangzhou Medical University from 2019.8.23 to 2021.1.23. The exclusion criteria were the following: co-infection with human immunodeficiency virus (HIV), syphilis, or other viral hepatitis, such as HCV and HDV.

Totally 421 individuals were selected, whose relevant data in the inspection system were collected, including pgRNA, HBV DNA, HBsAg, HBeAg, ALT, AST, PLT, etc. The above indicators were all derived from the detection of serum specimens. Through the medical record management system, information about basic characteristics and treatment were collected, such as age, gender, and treatment plan. The patient information involved has been replaced by initials and medical record numbers and would not involve patient privacy.

The baseline data were defined as the data when pgRNA was first detected within the study period. The data measured at the first follow-up after the baseline was retrospectively collected to conduct a retrospective cohort study.

This study was approved by the Ethics Committee of The Fifth Affiliated Hospital of Guangzhou Medical University.

### Detection of Serum Marker

Hepatitis B virus serum markers (HBsAg and HBeAg) were quantified by the Abbott ARCHITECT HBsAg and HBeAg, assays, respectively (detection limits: 1.00 s/co, 10.00 IU/L, 1.00 s/co, 1.00 s/co, and 1.00 s/co, respectively; Abbott Laboratories, Chicago, USA). The HBV DNA load was measured by a real-time PCR-based (detection limit: 100 IU/ml, Da'an Gene Co. Ltd., Sun Yat-Sen University, Guangdong, China).

pgRNA was detected by Hepatitis B Virus pgRNA Detection Kit (detection limit: 200 copies/ml, PCR-Fluorescent Probing, Guangzhou SUPBIO Biotechnology Co., Ltd., Guangdong, China), which used HBV-specific primers and probes (TaqMan probe), with PCR solution, thermostable DNA polymerase (Tth enzyme and H-Taq enzyme), reverse transcriptase (RT enzyme), RNase inhibitor, deoxyribonucleoside triphosphates (dNTPs), and other components. This kit was amplified by RT-PCR and used for the quantitative detection of HBV pgRNA in the sample. Internal control was set up to detect false negatives.

Reaction program of fluorescence quantitative PCR instrument:

Stage 1: 45°C 45 min, 95°C 2 min;

Stage 2: 95°C 15 s, 66°C 15 s, 72°C 20 s, repeat three times;

Stage 3: 95°C 15 s, 63°C 15 s, 72°C 20 s, repeat nine times;

Stage 4: 95°C 15 s, 60°C 45 s (fluorescence signal should be collected only in this step), repeat forty times;

FAM channel should be used for the detection of HBV pgRNA; VIC channel should be used for the detection of the internal control.

### The Settings of Variables and Concepts

1. pgRNA and HBV DNA: The pgRNA-negative was defined as the patients whose pgRNA were below the detection limit, namely, 200 copies/ml; the pgRNA-positive were defined as the patients whose pgRNA were higher than 200 copies/ml. So, HBV DNA was defined with 100 IU/ml as the detection limit.
2. HBeAg: Patients were divided into HBeAg-negative group (HBeAg < 1 COI/ml) and HBeAg-positive group (HBeAg >1 COI/ml) according to its clinical significance.
3. HBsAg: Patients were divided into a low HBsAg-level group and a high HBsAg-level group based on the median of the data in this study with 2,000 IU/ml as the cutoff point.
4. Age: Patients were divided into youth group (18–44 years) and non-youth group (>44 years) according to World Health Organization (WHO) standards for age classification.
5. Previous treatment plan: This variable was defined as the type of drug that had been taken by patients before the baseline.
6. FIB-4 (17): The index of FIB-4 was calculated as aspartate aminotransferase (AST) (IU/L) × age (years)/platelet count (10^9^/L)/alanine aminotransferase (ALT) (IU/L)^1/2^ for reflecting liver fibrosis and cirrhosis; those with FIB-4 <1.45 had no obvious liver fibrosis or only liver fibrosis below grade 2; those with FIB-4 >3.25 had liver fibrosis above grade 2.
7. Change magnitude: The change magnitude was defined as the numerical value of the second detection amount minus the baseline detection amount then divided by the baseline detection amount. The amounts of pgRNA for the second detection would be estimated at 100 copies/ml for the people that were negative in the second detection.
8. HBsAg significantly decreased: This concept was defined as the drop of HBsAg by one logarithmic value.
9. Treatment time: It is defined as the interval from baseline to the first follow-up visit.

### Statistical Analysis

The statistical analyses were performed by IBM SPSS statistics (version 25.0). Continuous variables were found non-normal by the one-sample Kolmogorov–Smirnov test and expressed as medians (inter-quartile ranges). Categorical variables were expressed as frequency (percentage). The variables were compared between groups using Mann–Whitney U and chi-square tests for univariate comparisons as well as the Kruskal–Wallis H test for multivariate comparisons. Logistic regression analysis was used to explore the influencing factors of pgRNA status. Linear regression analysis was used to explore the influencing factors of the change magnitude of pgRNA and HBsAg. Statistical significance was defined as *p* < 0.05 (two-tailed).

## Results

### Exploration of the Related Factors of pgRNA Status Based on Baseline Data

A total of 153 (36.3%) of the 421 subjects were pgRNA-negative and 268 (63.7%) were pgRNA-positive. Compared with the pgRNA-negative, the pgRNA-positive had statistically higher composition ratios of factors, including youth (85.8 vs. 65.4%), female (47.4 vs. 35.3%), positive HBV DNA (73.1 vs. 39.0%), high HBsAg level (60.4 vs. 38.8%), and positive HBeAg (76.1 vs. 3.6%). The compositions of the previous treatment plan before baseline were also statistically different between the different pgRNA groups, so were those of disease stage and FIB-4 grade (*p* < 0.05; Table 1).

**Table 1:** Description and difference analysis of factors under different pgRNA statuses.

**Factors**	**Total (%)**	**pgRNA-negative group (%)**	**pgRNA-positive group (%)**	***χ*^2^-value**	***P-*value**
	**421 (100)**	**153 (36.3%)**	**268 (63.7)**		
Age				24.067	<0.001
Youth	330 (78.4)	100 (65.4)	230 (85.8)		
Non-youth	91 (21.6)	53 (34.6)	38 (14.2)		
Gender				5.812	0.016
Male	240 (57.0)	99 (64.7)	141 (52.6)		
Female	181 (43.0)	54 (35.3)	127 (47.4)		
Disease stage				9.833	0.020
Carriers of HBsAg	129 (30.6)	49 (32.0)	80 (29.9)		
Chronic hepatitis B	226 (53.7)	70 (45.7)	156 (58.2)		
Liver cirrhosis	29 (6.9)	16 (10.5)	13 (4.8)		
Other	37 (8.8)	18 (11.8)	19 (7.1)		
Previous treatment plan				20.960	<0.001
None	206 (48.9)	74 (48.4)	132 (49.3)		
Tenofovir	109 (25.9)	24 (15.7)	85 (31.7)		
Entecavir	85 (20.2)	45 (29.4)	40 (14.9)		
Other treatment plans	21 (5.0)	10 (6.5)	11 (4.1)		
HBV DNA				34.870	<0.001
Negative	122 (38.0)	64 (61.0)	58 (26.9)		
Positive	199 (62.0)	41 (39.0)	158 (73.1)		
HBsAg				13.673	<0.001
Low HBsAg level	149 (47.6)	71 (61.2)	78 (39.6)		
High HBsAg level	164 (52.4)	45 (38.8)	119 (60.4)		
HBeAg				184.104	<0.001
Negative	190 (50.0)	132 (96.4)	58 (23.9)		
Positive	190 (50.0)	5 (3.6)	185 (76.1)		
FIB-4 grade				9.219	0.010
Below grade 2	228 (83.2)	79 (75.2)	149 (88.2)		
Grade 2	23 (8.4)	11 (10.5)	12 (7.1)		
Above grade 2	23 (8.4)	15 (14.3)	8 (4.7)		

*HBV, hepatitis B virus; HBsAg, hepatitis B surface antigen; HBeAg, hepatitis B e antigen*.

Patients with tenofovir treatment were more likely to be pgRNA-positive (OR: 23.47, 95%CI: 3.35–164.65), so were those with entecavir treatment (OR: 14.90, 95%CI: 2.18–101.90) (*p* < 0.05). What is more, positive HBV DNA (OR: 40.51, 95% CI: 7.00–234.55) was an independent risk factor of positive pgRNA, so was positive HBeAg (OR: 66.24, 95%CI: 20.03–219.07) (*p* < 0.05). Conversely, there was no statistical significance of factors, including age (OR: 1.14, 95% CI: 0.46–2.81), gender (OR: 1.43, 95%CI: 0.64–3.19), and other treatment plans (OR: 3.81, 95%CI: 0.46–31.54) (*p* > 0.05; Table 2).

**Table 2:** Multivariate logistic regression analysis results of baseline pgRNA influencing factors.

**Factors**	***B***	***Wald* value**	***p*-value**	**Odds ratio (95%CI)**
Constant	−4.517	12.764	<0.001	0.01
Age	0.131	0.080	0.777	1.14 (0.46, 2.81)
Gender	0.356	0.753	0.386	1.43 (0.64, 3.19)
Previous treatment plan		10.715	0.013	
Tenofovir	3.156	10.083	0.001	23.47 (3.35, 164.65)
Entecavir	2.702	7.586	0.006	14.90 (2.18, 101.90)
Other treatment plans	1.338	1.541	0.215	3.81 (0.46, 31.54)
None	Control			
HBV DNA	3.701	17.064	<0.001	40.51 (7.00, 234.55)
HBeAg	4.193	47.214	<0.001	66.24 (20.03, 219.07)

### Exploration of the Clinical Significance Between pgRNA and Liver Fibrosis on Baseline Data

**Figure 1:**
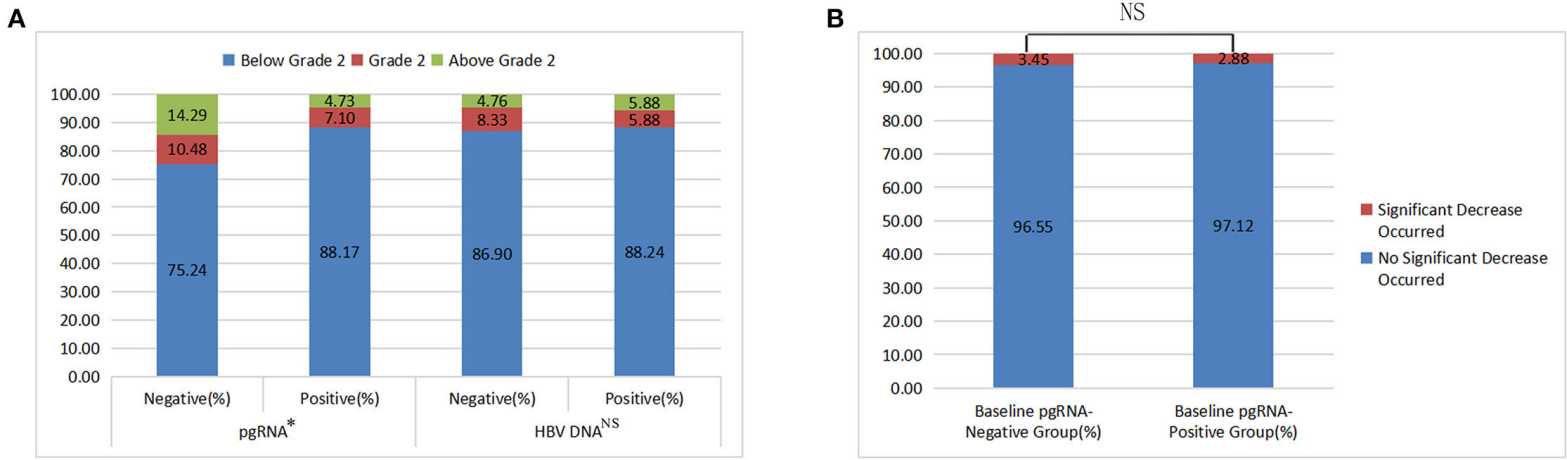
The results of chi-square tests (**A**: FIB-4 grade among different baseline pgRNA and HBV DNA groups; **B**: incidence of a significant decrease in HBsAg between different baseline pgRNA groups).

As mentioned above, the compositions of FIB-4 grade were statistically different between different pgRNA groups (*p* < 0.05; Table 1). Number of patients with FIB-4 below grade 2, grade 2, and above grade 2 were 73, 7, and 4, respectively, in the baseline HBV DNA-negative group, and 105, 7, and 7, respectively, in the baseline HBV DNA-positive group. As shown in Figure 1A, there was no statistical difference between different HBV DNA groups (χ^2^ = 0.553, *p* = 0.758).

As shown in Table 3, in the baseline, the difference in the FIB-4 index was statistically significant between different groups of pgRNA status. Conversely, that was not statistically significant between different groups of HBV DNA status.

**Table 3:** Description and difference analysis of FIB-4 index among different baseline pgRNA and HBV DNA groups.

**Group**	**P25**	***M***	**P75**	***Z*-value**	***p-*value**
Total	0.404	0.677	1.109	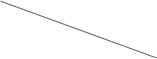	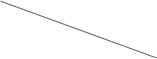
HBV DNA				−1.041	0.298
Negative	0.488	0.682	0.882		
Positive	0.376	0.605	1.070		
pgRNA				−3.479	0.001
Negative	0.474	0.757	1.453		
Positive	0.330	0.581	0.913		

Obviously, whether FIB-4 grades or FIB-4 indexes were statistically different between different pgRNA groups, while not statistically different between different HBV DNA groups, suggesting that pgRNA had a better correlation with liver fibrosis than HBV DNA.

### Exploration of the Influencing Factors of pgRNA Change Magnitude Based on a Retrospective Cohort Study

At last, totally 89 patients who had detected pgRNA two times or more times were included, among which 20 (22.5%) were negative for baseline pgRNA. Among the remaining 69 (77.5%), 7 (10.1%) were pgRNA-negative in the second detection. Because of the calculation of the change magnitude, it is obvious when the change magnitude was negative, the greater the absolute value, the greater the decline in pgRNA.

**Figure 2:**
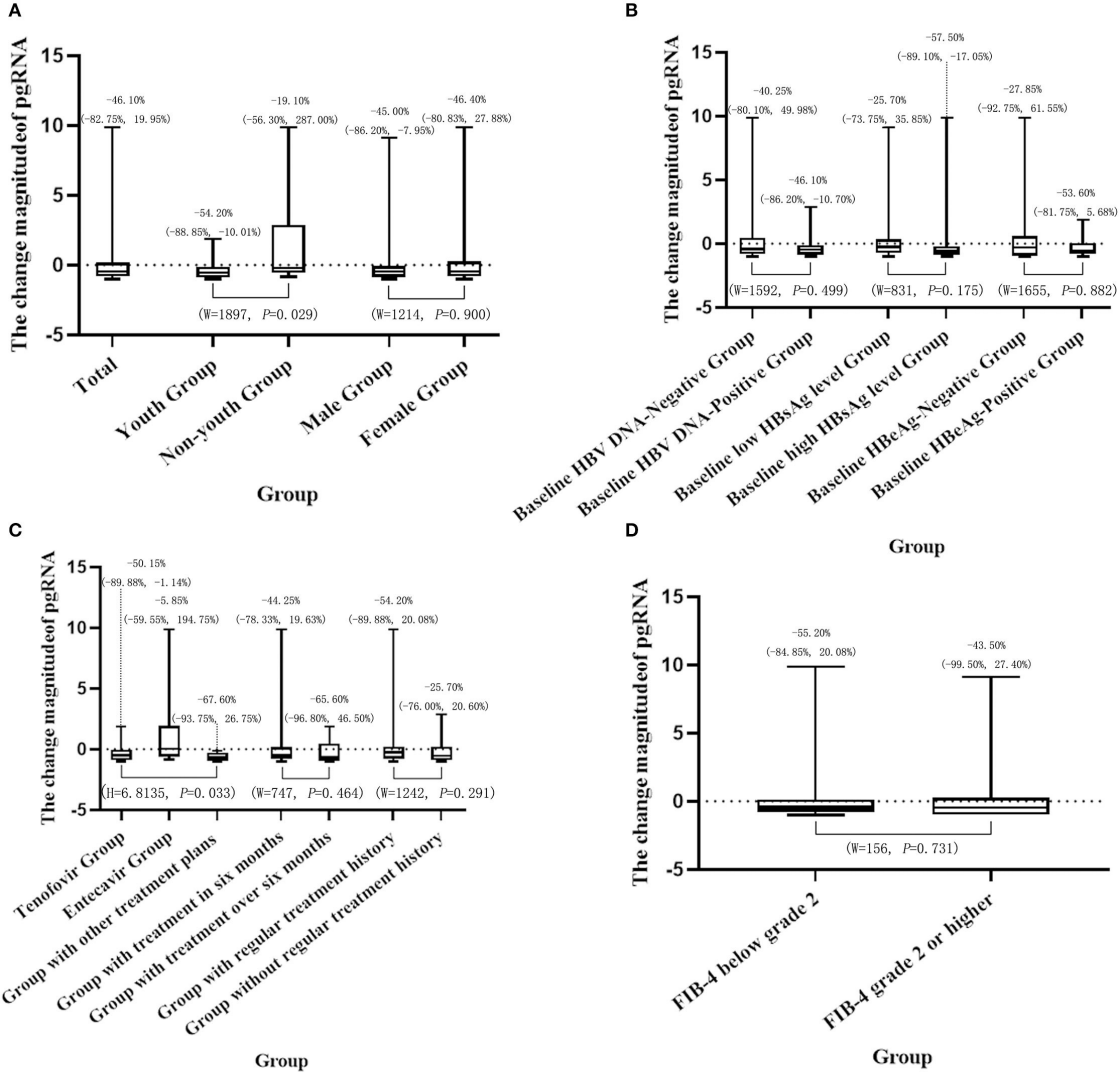
Description and results of difference analysis of the pgRNA change magnitude in different groups (**A**: general characteristics; **B**: HBV serum marker status; **C**: treatment situation; **D**: degree of liver fibrosis).

There was a significant difference in pgRNA change magnitude between different age groups, so was there among different treatment plan groups (*p* < 0.05). However, there was no significant difference in pgRNA change magnitude between different groups of other factors, including gender, treatment time, baseline HBV DNA status, baseline HBsAg status, baseline HBeAg status, FIB-4 classification, and history of formal antiviral therapy (*p* > 0.05). It is worth mentioning that 46 (51.7%) patients treated for <6 months had no significant difference in the pgRNA change magnitude compared with 43 (48.3%) patients treated for more than 6 months (*p* > 0.05; Figure 2).

Univariate linear regression of treatment plan showed that compared with the patients treated with tenofovir, the patients treated with entecavir (B: 1.806, 95%CI: 0.777–2.835) had higher pgRNA change magnitude. The conclusion could be drawn that the decline in pgRNA of patients treated with tenofovir was greater than that of patients treated with entecavir. There was no statistical significance in other treatment plans (B: −0.285, 95%CI: −1.514–0.944) (Table 4).

**Table 4:** The results of linear regression about pgRNA change magnitude.

**Factors**	***B*(95%CI)**	**Beta**	***t*-value**	***p*-value**
**Univariate linear regression about the influence treatment plan exert on pgRNA change magnitude**				
Constant	−0.305 (−0.802, 0.192)		−1.225	0.225
Tenofovir	Control			
Entecavir	1.806 (0.777, 2.835)	0.400	3.503	0.001
Other treatment plans	−0.285 (−1.514, 0.944)	−0.053	−0.463	0.645
**Multivariate linear regression about influencing factors of pgRNA change magnitude**				
Constant	−2.307 (−4.308,−0.306)		−2.303	0.025
Age	1.588 (0.268, 2.907)	0.320	2.404	0.019
Gender	0.204 (−0.592, 1.000)	0.056	0.513	0.610
**Treatment plan**				
Tenofovir	Control			
Entecavir	1.021 (−0.181, 2.223)	0.226	1.697	0.094
Other treatment plans	−0.166 (−1.366, 1.035)	−0.031	−0.276	0.784

Multivariate linear regression showed age (B: 1.588, 95%CI: 0.268–2.907) was an independent factor of pgRNA change magnitude. In other words, compared with young patients, non-young patients had higher pgRNA change magnitude. Therefore, the decline in pgRNA of young patients was greater than that of non-young patients. The multivariate linear regression model showed no statistically significant relationship of gender or treatment plan (*p* > 0.05; Table 4).

### Exploration About the Influence of pgRNA on HBsAg Change Magnitude Based on Retrospective Cohort Study

At last, totally 133 patients who had baseline pgRNA data and had detected HBsAg two times or more were included. As shown in Figure 1B, there was no statistical difference in the incidence of “HBsAg significantly decreased” between different pgRNA groups (χ^2^ = 0.025, *p* = 0.875). Besides, as shown in Table 5, the difference in HBsAg change magnitude was also not statistically significant between different groups of baseline pgRNA status (*Z* = −1.003, *p* = 0.316).

**Table 5:** Description and difference analysis of HBsAg change magnitude between different baseline pgRNA groups.

**Group**	**P25**	***M***	**P75**	***Z-*value**	***p-*value**
Total	−37.74%	−13.65%	3.69%	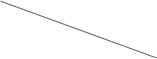	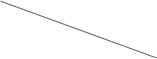
Baseline pgRNA				−1.003	0.316
Negative	−36.62	−15.19	−5.08		
Positive	−37.80	−10.63	5.78		

The conclusion could be drawn that no statistical difference was found between different baseline pgRNA groups by calculating the incidence of the significant decrease in HBsAg or HBsAg change magnitude. In other words, the status of baseline pgRNA was not statistically related to the change of HBsAg during treatment.

## Discussion

Chronic hepatitis B is still one of the major infectious diseases in the world. Previous studies on HBV pgRNA mainly focus on the molecular level, while not many large-scale studies focus on the public level.

**Figure 3:**
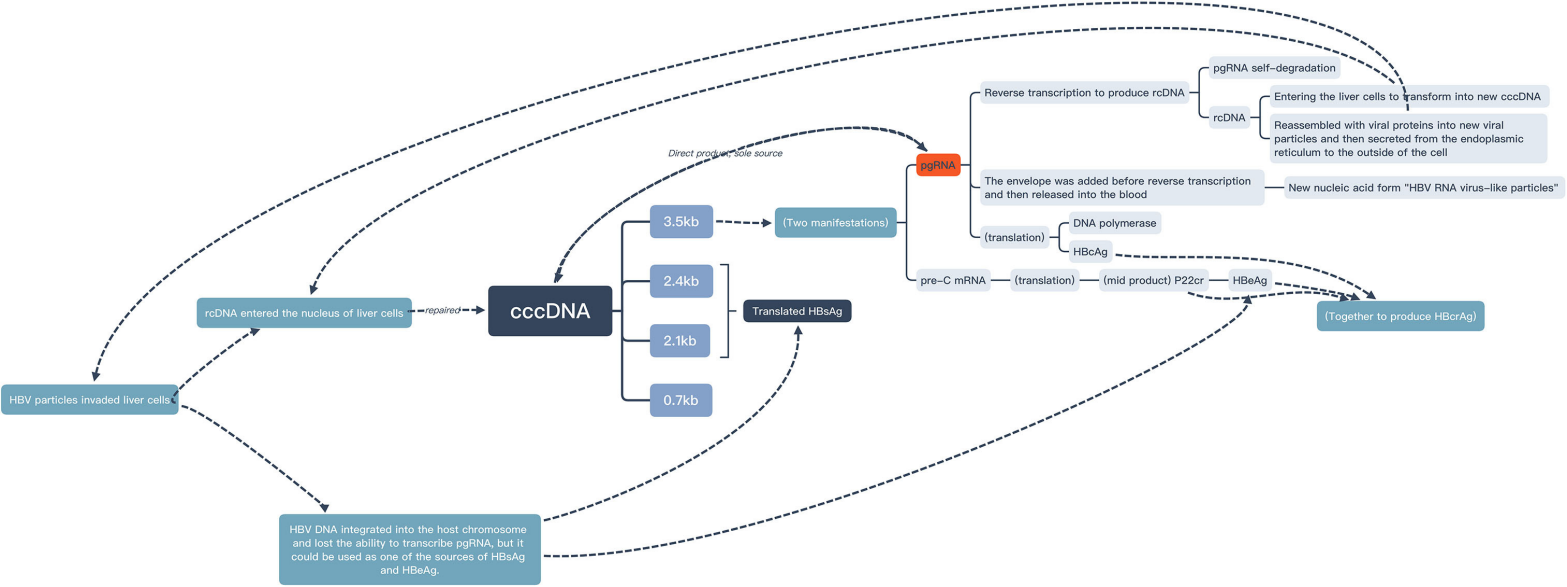
Source and fate of pgRNA and related substances (2, 4, 20, 23–25).

Positive HBV DNA was found a risky factor of positive pgRNA. This was consistent with the conclusions drawn by other population studies (18, 19). From a molecular perspective, HBV DNA in serum was mainly derived from the reverse transcription of HBV pgRNA (4, 20) (Figure 3), which could also support this conclusion.

Positive HBeAg was also found a risky factor of positive pgRNA. This was the same conclusion reached by other population studies (4, 21, 22). From a molecular perspective, HBeAg was derived from pre-C mRNA which was transcribed from HBV cccDNA. Like pgRNA, pre-C mRNA was one of the manifestations of 3.5 kb RNA transcribed from HBV cccDNA (23) (Figure 3). It was speculated that the two kinds of 3.5 kb RNA were transformed according to a certain ratio. In other words, there was a high load of HBV cccDNA and a high degree of HBV virus expression in the liver of patients in the active phase.

This study suggested that patients who had received regular antiviral therapy with tenofovir or entecavir before detection were more likely to have positive pgRNA than patients who had not had received regular antiviral therapy. This was consistent with the conclusion of this study (24), which showed that HBV pgRNA transcribed from HBV cccDNA would be degraded during reverse transcription, whereas NAs drugs could inhibit the reverse transcription process, resulting in the accumulation and increase in HBV pgRNA in the serum of patients receiving NAs treatment (Figure 3). Moreover, Pan (26) found that after 5 years of NAs treatment, 45% of the patients were pgRNA-positive, which showed that even after long-term antiviral therapy, pgRNA can still reflect the activity of cccDNA. The cccDNA transcriptional activity of patients who were still pgRNA-positive after antiviral therapy for many years might be higher than that of other patients.

This study found that the pgRNA-positive group had significantly lower FIB-4 grade and FIB-4 index than the pgRNA-negative group, which might be related to the decrease in HBV replication caused by liver cell damage when the degree of liver fibrosis increased (27). Besides, the conclusion that FIB-4 was significantly related to pgRNA status, but not to HBV DNA, reminded us that in clinical work, especially for liver fibrosis, in addition to paying attention to HBV traditional indicators, such as HBV DNA, we should also focus on HBV pgRNA.

This study found that age was an independent factor of pgRNA change magnitude. Young patients had a greater decline in pgRNA than non-young patients. This might be related to young people's stronger immunity, more active organ functions, better liver cell regeneration, fewer complications, and so on.

The univariate linear regression found that patients treated with tenofovir were more likely to get pgRNA decrease than patients treated with entecavir. In addition, the *p-*value was also <0.1 in the multivariate regression. This suggested that tenofovir's ability to reduce pgRNA may be more superior than entecavir's. Similar results of other HBV markers, such as HBV DNA (28, 29) and HBsAg (30), were found in some studies. The speculation could be conclusively made that compared with entecavir, tenofovir treatment may be able to make the HBV cccDNA in the patient's liver decline faster and hepatitis B recurrence less likely; however, further research studies were needed to confirm the speculation.

We did not find any significant correlation between pgRNA and HBsAg by calculating whether the categorical variable “whether HBsAg significantly decreased” or the quantitative variable “HBsAg change magnitude”. This was consistent with the results of the cross-section of this study. However, due to the small sample size and short follow-up time, the possibility of false negatives cannot be ruled out. In addition, referring to the definition of “low viral load” in the study (31) (positive HBsAg but negative HBeAg) and the guidelines (1) (HBV DNA ≤ 2,000 IU/ml), in this retrospective cohort study, 50 (37.6%) patients were HBeAg-negative, and 62 (46.6%) patients had HBV DNA lower than 2,000 IU/ml, who could be classified as patients with low viral load. Studies (10, 32) had found that when the virus in patients was in a state of low expression, a considerable proportion of HBsAg was derived from HBV DNA integrated into the chromosome of the host liver cell. At this time, the HBsAg level was less affected by the pgRNA level, and the relationship between HBsAg and pgRNA also disappeared (33) (Figure 3). The statistical difference might be able to be observed in the case with a larger sample and a longer observation period.

In summary, this study described the status of pgRNA and analyzed its influencing factors, verified the significant correlation between pgRNA and HBeAg as well as HBV DNA, pointed out the relevance of pgRNA in liver fibrosis, enriched the clinical significance of pgRNA, and found that antiviral therapy could also affect the status of pgRNA. Besides, this article studied the influencing factors of pgRNA change magnitude and further explored the influence of age and antiviral treatment plans on pgRNA change magnitude, which had certain guiding significance for clinical medication.

It is undeniable that this study also had some shortcomings. This study was a retrospective study conducted in a single hospital, which could not represent the entire population well. As a result, the extrapolation ability was not strong. In addition, Wang et al. (16) found that pgRNA rather than pre-C mRNA was the only HBV RNA in the serum of patients with chronic hepatitis B; however, in 2020, Prakash et al. (35). used supersensitive droplet digital PCR and found that most of HBV RNA in serum was pgRNA, but a tiny amount was pre-C RNA. Both pre-C RNA and pgRNA belong to 3.5kb HBV RNA (34), but pre-C RNA was slightly longer than pgRNA (35). This made it difficult to design specific primers for pgRNA. Therefore, the level of detected pgRNA may be higher than the true value due to the inclusion of a small amount of pre-C RNA. Although the levels of pre-C RNA were ~2 log_10_ units lower than those of pgRNA (35) and might have a limited impact on the result, it may be more appropriate to call the “pgRNA” in this study “HBV RNA”.

## Data Availability Statement

The raw data supporting the conclusions of this article will be made available by the authors, without undue reservation.

## Ethics Statement

The studies involving human participants were reviewed and approved by the Human Ethics Committee of The Fifth Affiliated Hospital of Guangzhou Medical University. The patients/participants provided their written informed consent to participate in this study.

## Author Contributions

H-ZY, SO-Y, Z-HH, and X-GG developed the concept of the study. L-LY, C-WL, and T-TP participated in its design and coordination and helped draft the manuscript. H-ZY, T-TP, and X-GG contributed to the acquisition and interpretation of data. SO-Y provided a critical review and substantially revised the manuscript. All authors read and approved the final manuscript.

## Funding

This study was supported in part by grants from the National Natural Science Foundation of China (Grant No. 81803884), the Natural Science Foundation of Guangdong Province, China (Grant No. 2015A030313684), the Scientific Research Project of Guangdong Provincial Bureau of Traditional Chinese Medicine (Grant No. 20191215), and Guangzhou Key Laboratory Fund (Grant No. 201905010004).

## Conflict of Interest

The authors declare that the research was conducted in the absence of any commercial or financial relationships that could be construed as a potential conflict of interest.

## Publisher's Note

All claims expressed in this article are solely those of the authors and do not necessarily represent those of their affiliated organizations, or those of the publisher, the editors and the reviewers. Any product that may be evaluated in this article, or claim that may be made by its manufacturer, is not guaranteed or endorsed by the publisher.

## Abbreviations

pgRNA: pregenomic RNA
HBV: hepatitis B Virus
CHB: chronic hepatitis B
cccDNA: covalently closed circular DNA
rcDNA: relaxed circular DNA
AASLD: American Association for the Study of Liver Diseases
EASL: European Association for the Study of the Liver
HBsAg: hepatitis B surface antigen
HIV: human immunodeficiency virus
HCV: hepatitis C Virus
HDV: hepatitis D Virus
HBeAg: hepatitis B e antigen
ALT: alanine aminotransferase
AST: aspartate aminotransferase
PLT: platelet count
PCR: polymerase chain reaction
RT: reverse transcriptase
dNTPs: deoxyribonucleoside triphosphates
NAs: nucleos(t)ide analog.
